# Burden of injury in childhood and adolescence in 8 European countries

**DOI:** 10.1186/1471-2458-10-45

**Published:** 2010-01-29

**Authors:** Suzanne Polinder, Juanita A Haagsma, Hidde Toet, Marco JP Brugmans, Ed F van Beeck, the EUROCOST and APOLLO reference groups

**Affiliations:** 1 Department of Public Health, Erasmus Medical Centre, University Medical Centre Rotterdam, The Netherlands; 2 Consumer Safety Institute, Amsterdam, The Netherlands

## Abstract

**Background:**

Injury is the major cause of death and suffering among children and adolescents, but awareness of the problem and political commitment for preventive actions remain unacceptably low. We have assessed variation in the burden of injuries in childhood and adolescence in eight European countries.

**Methods:**

Hospital, emergency department, and mortality databases of injury patients aged 0-24 years were analyzed for Austria, Denmark, Ireland, Latvia, Netherlands, Norway, Slovenia and the United Kingdom (England, Wales). Years lost due to premature mortality (YLL), years lived with disability (YLD), and disability adjusted life years (DALYs) were calculated.

**Results:**

Differences in the burden of injury in childhood and adolescence are large, with a fourfold gap between the safest countries (Netherlands and UK) in western-Europe and the relatively unsafe countries (Latvia and Slovenia) in the east. Variation between countries is attributable to high variation in premature mortality (YLL varied from 14-58 per 1000 persons) and disability (YLD varied from 3-10 per 1000 persons). Highest burden is observed among males ages 15-24. If childhood and adolescence injuries are reduced to the level of current best injury prevention practices, 6 DALYs per 1000 child years can be avoided.

**Conclusions:**

Injuries in childhood and adolescence cause a high disability and mortality burden in Europe. In all developmental stages large inequalities between west and east are observed. Potential benefits up to almost 1 million healthy child years gained across Europe are possible, if proven ways for prevention are more widely implemented. Our children deserve action now.

## Background

All over the globe, injuries in childhood and adolescence have a major impact on individual and population health. Each day, the ideals of thousands of our vivid and promising youth are suddenly destroyed by road traffic crashes, injuries at home or during leisure time, or acts of violence. This is largely unnecessary, since an abundance of simple and effective countermeasures are available (e.g. use of bicycle helmets, reduced speed limits, barrier fencing on swimming pools, lower tap water temperatures), but these are underused[1]. For this reason, even in high-income countries, injury is still the leading cause of death and disability among children and adolescents[2,3]. In Europe, each year more than 40,000 children die from injury, [4] and for every child fatality, there are several thousand victims of injury or violence who live with varying degrees of disability or psychological scarring[1]. According to WHO, "awareness of the problem and its preventability, as well as political commitment to act to prevent child injury, remain unacceptably low"[1]. Therefore, as a first step in addressing this problem, among health policy makers awareness should be raised and priority areas with the highest potential health gains should be identified.

This can be accomplished by providing decision makers with summary measures of population health, such as the disability-adjusted life year (DALY)[5]. The DALY is the sum of years lost due to premature mortality (YLL) and years lived with disability (YLD). A high number of DALYs reflects a greater burden of death and disability. The DALY was aimed at national and international health policies, to develop unbiased epidemiological assessments for major disorders, and to provide an outcome measure that could also be used for cost-effectiveness analysis[6].

The human impact of childhood and adolescence injury in terms of DALYs in Europe by country, age, sex, injury type and external cause specifically has not been addressed yet. Expected variation in the burden of injury among the European countries may be caused by differences in exposure, injury risk and type of sustained injury, differences in demography, (socio)economic and cultural factors, safety technology, injury-prevention strategies, and the effectiveness of trauma care. Assessment of the variation of the burden of injury and its separate components can be used to identify high-risk groups in Europe as well as in specific European countries, and it can be used to prioritise injury-prevention programmes.

In this paper we assess the impact and variation in the burden of injury in childhood and adolescence in eight European countries. This is expressed in the summary measure of DALYs and its components, namely premature mortality (years of life lost, YLL) and disability (years lived with disability, YLD). To show potential health gains, we estimate the impact on mortality and (life-long) disability if childhood and adolescence injuries were reduced to the level of current best injury prevention practices in Europe.

## Methods

### General approach

Data collection and analysis were conducted within European collaborative efforts on standardisation of methods to support injury prevention policies, namely the EUROCOST [7] and the APOLLO [8] project. We compared the number of DALYs attributable to unintentional and intentional injuries of patients in the age of 0-24 years in eight European countries. The incidence data of Austria, Denmark, Ireland, Netherlands, Norway, and the United Kingdom (England and Wales) were supplied by EUROCOST project, whereas the APOLLO project provided opportunities to include data from two countries that only recently (2004) joined the European Union, i.e. Latvia and Slovenia. Comparative data on the burden of injury in Europe not specified by age category and without the latter two countries, were published elsewhere[9]. Comparable data sources in other European countries were either unavailable or could not be collected and analysed within the framework used. We used two primary data sources: 1) hospital discharge registers (HDR) with full national coverage to estimate the hospitalization rate and 2) emergency-department (ED) surveillance systems for the incidence of non-admitted ED patients[10,11]. Since ED systems did not have nationwide coverage, country-specific extrapolation factors were used to extrapolate the ED incidence towards national level for the respective types of injury by country[7,11]. We computed YLL using a standard life table[5,12]. YLD were obtained by multiplying frequency, duration and injury-specific severity weights of the injury. DALYs were the summation of YLLs and YLDs[5].

### Incidence and mortality data

We used the International Classification of Disease codes 800 to 999 (ICD, 9th revision) [13] and corresponding codes of ICD-10 for countries that used this revision to select and classify both unintentional and intentional injuries. Following international recommendations, we excluded 'misadventures to patients during surgical and medical care' (ICD-9 E996-999, E870-E876), 'surgical and medical procedures as the cause of abnormal reaction of patients or later complication, without mention of misadventure at the time of procedure' (ICD-9 E878-E879), 'drugs, medicaments and biological substances causing adverse effects in therapeutic use' (ICD-9 E930-E949), and late effects of injury (ICD-9 E905-E909)[14].

Table 1 provides an overview of the incidence and mortality data by country. Non-admitted injury patients included in the study were derived from ED systems and hospitalized patients were derived from HDR. For Latvia, the Netherlands, Norway, Slovenia and UK Wales, the ED surveillance system comprised all types of injuries, whereas for Denmark it was restricted to all unintentional injuries. For Austria, Ireland, and UK England only to home and leisure injuries were registered in the ED surveillance system. Home and leisure injuries account for 70-78% of ED visits in the five countries with all full injury data available.

**Table 1:** Incidence and mortality due to injury per 1,000 persons in the age group 0-24 years, by country: absolute numbers and rates

Country	Absolute numbers			Per 1,000 inhabitants		
	
	Incidence		Deaths ^c^	Incidence		Mortality rate ^c^
				
	Not-admitted ED^a ^patients	Hospitalized patients^b^		Not-admitted ED^a ^patients	Hospitalized patients ^b^	
Austria ^d^	201 944^f^	51 819	665	85.9	22.0	0.29
Denmark ^d^	290 439^g^	29 396	371	181.6	18.4	0.23
Ireland ^d^	76 502^d^	25 882	355	51.7	17.5	0.24
Latvia ^e^	177 204	13 985	488	256.3	20.2	0.71
Netherlands^d^	468 660	28 759	704	97.0	6.0	0.15
Norway^d^	188 455	17 510	385	128.6	12.2	0.27
Slovenia ^e^	5 544	8 405	204	36.0^h^	15.4	0.37
UK, England^d^	309 1943^f^	235 498	3175	200.0	15.2	0.19
UK, Wales ^d^	156 682	17 354		171.7	19.0	

^a ^ED = Emergency department; data extrapolated.

^b ^Data from hospital discharge registers.

^c ^Data from World Health Organization Mortality Database 2005.

^d ^ED and HDR incidence data from the year 1999 (EUROCOST project).

^e ^ED and HDR incidence data from the year 2005 (APOLLO project).

^f ^Only home and leisure injury data included.

^g ^Only unintentional injury data included.

^h ^Incidence rate of non-admitted ED patients in Slovenia is an underestimation, due to an incorrect extrapolation factor.

For the mortality data, we used age- and sex-specific death rates from the WHO mortality database for the year 2005[15]. This database includes information on the external cause, whereas information on injury diagnosis is not usually available.

### Calculation of YLD and YLL

YLD was obtained by multiplying the incidence of cases of injury (both hospitalized and non-admitted ED) by the average duration, based on the weights per injury group as recommended in the global burden of disease (GBD) study, performed at the request of WHO, and by a disability weight. Disability weights are values that represent the severity of health status associated with specific diseases and injuries[5]. The GBD disability weights and our data sources were compatible for 30 injury groups (see Additional file 1). Burns were excluded from the analyses since our data did not specify the percentage surface area burned and/or severity of the wounds, while wound severity is a major determinant for recovery duration and disability.

The GBD determined a comprehensive set of short-term (first year after injury) and lifelong sequelae. For hospitalized patients, for certain injuries the GBD formulated predefined proportions of life-long disability (see Additional file 1)[5]. Durations of permanent disability were estimated by multiplying the incidence by the age- and sex-specific life expectancies, derived from the standard life table used in the GBD study (West Level 26 life-table)[5]. It was assumed that non-admitted ED patients suffered short-term disability only. There were no GBD disability weights available for concussion, whiplash, and superficial injury. To reduce underestimation of short-term YLD, for these health states we adopted disability weights from a set of novel disability weights, recently developed in the Netherlands[16]. These disability weights were derived from health state valuations of a population panel, applying a renewed methodology that focused especially on obtaining and improving injury disability weights for temporary functional losses. In industrialized countries the majority of patients with eye injury have only minor temporary problems [17], therefore we adopted the disability weight for corneal abrasion from the Dutch set of disability weights as well[16].

To avoid double counting with YLL, the fraction of hospitalised injury patients that died in hospital was excluded from the YLD calculations.

YLL were calculated using the West Level 26 life-table and estimated mean age at death by age category [12]. The West Level 26 life-table has a standard life expectancy at birth, 80.0 years for males and 82.5 years for females. To yield YLL due to injury, standard YLL were multiplied by mortality rates and population numbers. Age-weights or discounting were not applied in the calculations, because this practice is considered controversial[18,19].

## Results

### DALYs by country

Marked differences existed in the burden of childhood and adolescent injury (table 2), with a fourfold gap between Netherlands and UK (respectively 14 and 18 DALYs per 1000 persons) and Latvia (58 DALYs per 1000 persons). Denmark, Ireland, and Norway have rather comparable estimates varying between 22 and 24 DALYs per 1000 persons. Austria lost a relatively large number of DALYs compared to the other 'Western European countries' due to the highest YLD loss caused by life-long injury and a high mortality (twice the mortality rate of the Netherlands, see Table 1). The variation in the burden of injury for persons in the age of 0-24 years between the countries, as shown in Table 2, is primarily a reflection of an enormous variation in premature mortality (YLL varied from 11-52 per 1000 persons), although YLD also shows variation between countries (variation from 2-10 YLD). In all participating countries, the largest part of the total burden (67-89%) was caused by premature mortality. The burden due to permanent (life-long) disability was high compared to temporary (short-term) disability.

**Table 2:** Disability (YLD), premature mortality (YLL), and burden of injury (DALY) per 1,000 persons in the age group 0-24 years, by country

Country	Disability				Premature mortality	Burden of injury
	
	YLD^a^	YLD^a^	YLD^a^	YLD^a^	YLL^b^	DALY^c^
	Not admitted short term	Admitted short term	Admitted life long	Total		
Austria	0.9	0.3	9.2	10.4	20.8	31.2
Denmark	1.3	0.2	3.6	5.1	16.8	21.9
Ireland	0.4	0.1	5.7	6.2	17.2	23.4
Latvia	2.0	0.2	4.4	6.6	51.6	58.2
Netherlands	0.8	0.1	2.2	3.1	10.7	13.8
Norway	1.0	0.2	3.1	4.3	19.4	23.7
Slovenia	0.1	0.2	2.1^d^	2.4^d^	27.0	29.4
UK, England	0.8	0.1	2.9	3.8	14.0	17.8
UK, Wales	1.1	0.1	2.8	4.0	14.0	18.0

^a ^YLD = years lived with disability

^b ^YLL = years lost due to premature mortality

^c ^DALY = disability-adjusted life years

^d ^YLD admitted life long and YLD total are an underestimation, since spinal cord injury and complex soft tissue injury are not coded in the HDR.

### DALYs by age and sex

**Figure 1:**
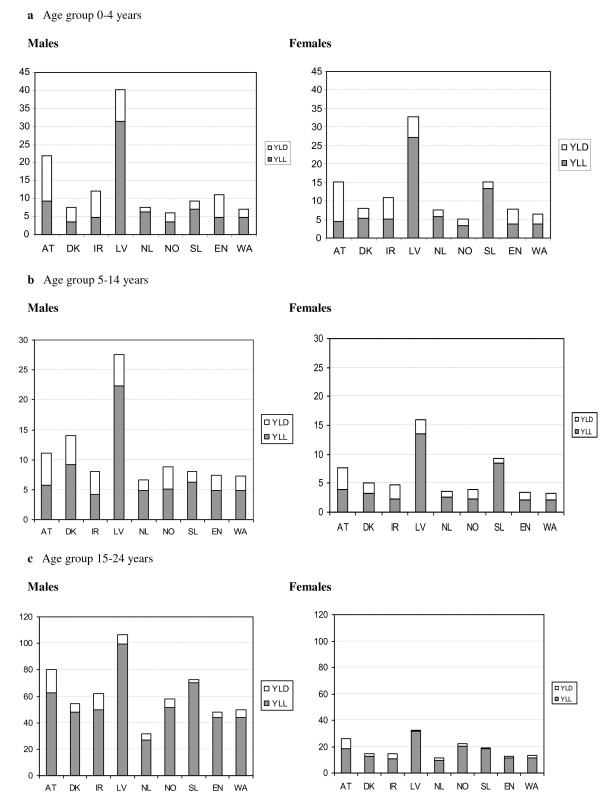
**The burden of injury as DALYs per 1000 persons, divided into YLD and YLL**. YLD = years lived with disability; YLL = years lost through premature mortality. AT = Austria; DK = Denmark; IR = Ireland; LV = Latvia; NL = Netherlands; NO = Norway; SL = Slovenia; EN = UK, England; WA = UK, Wales.

Figure 1 gives an overview of the total DALYs (separated into YLL and YLD) by age and sex for all participating countries. For children aged 0-4 years the burden of injury among boys and girls is quite comparable. Within this youngest age group, the burden related to disability contributes for around 30% to total disease burden for both boys and girls, which is mainly the consequence of skull-brain injury.

In most countries, the burden of injury is lowest in the age group of 5-14 year old children and the burden of premature mortality is in general twice as high for boys as for girls. For all countries, in males aged 15-24 years by far the highest number of DALYs per 1000 persons is observed, which is caused by high premature mortality (YLL). In this age group, the burden of premature mortality is approximately three times higher for males than for females. Among European countries, there are striking differences in total DALYs due to injuries by age and sex. Noteworthy is the high mortality burden for boys and girls in all age groups in Latvia compared to the other countries. The Netherlands, UK England, and UK Wales show a relatively low burden of injury across all age groups and for both sexes.

### YLD by injury group

The injury burden by injury group incorporates disability only, because injury-specific mortality data were not available (Table 3). In a pooled analysis of data of all participating countries, skull-brain and spinal cord injury resulted in by far the highest total YLD due to life-long disability for the majority of the young children and adolescents. The disability burden of skull-brain injury appears nearly equivalently distributed among all age groups for most countries. On the other hand, the high disability burden related to spinal cord injury is primarily caused by young adults in the age of 15-24 year, mainly due to traffic accidents in males. Superficial injury resulted in the highest short-term disability, due to a high ED and clinical incidence.

**Table 3:** Clinical incidence and disability (YLD) due to injury per 100,000 persons in the age group 0-24 years, by injury group (ranked by total YLD)

Rank	Injury	Clinical incidence^a ^(ED visits)	Disability		
			
			YLD short-term	YLD lifelong	YLD total
1	Skull--brain	53.1 (197.6)	6.0	185.8	191.8
2	Spinal cord	3.2	^b^	140.5	140.5
3	Superficial injury	118.6 (3351.1)	32.4	^c^	32.4
4	Injury of nerves arm/hand	7.8	^b^	31.8	31.8
5	Complex soft tissue injury upper extremity	34.2	^b^	30.2	30.2
6	Fracture of femur shaft	18.8 (36.6)	2.1	17.6	19.8
7	Complex soft tissue injury lower extremity	7.6	^b^	11.9	11.9
8	Fracture of elbow/forearm	89.2 (466.0)	8.5	^c^	8.5
9	Fracture wrist (incl. carpal bones)	59.3 (462.8)	6.0	^c^	6.0
10	Concussion	170.9 (189.3)	5.4	^c^	5.4

^a ^From hospital discharge registers.

^b ^All patients have life long disability.

^c ^All patients have short-term disability.

### Preventable burden of injury in the European Union

An estimation was made of the impact on mortality and (life-long) disability if childhood and adolescence injuries were reduced to the injury safety level of the Netherlands, which was interpreted as the current best injury prevention practice in Europe. Therefore, we recalculated the burden of injury by substituting the YLD and YLL outcomes of the Netherlands (per 1,000) on the population deviation of the other countries. When only the seven other described countries in this study are taken into account, the potential health gains would be 197,000 avoided DALYs (42,000 YLD and 155,000 YLL) for the total number of children and adolescents (32 million children and adolescents in the seven countries, 6 per 1,000).

Furthermore, we have calculated the potential health gains regarding childhood and adolescence injuries in the European Union (EU, 27 countries), assuming that the Netherlands has the current best injury prevention practice in the EU (which is supported by other studies [4]) and assuming that the other described countries in this study are representative for the EU countries not included in this study. Under these assumptions, the potential achievements reached by preventing injuries in the EU amounts to almost 900,000 DALYs avoided per 140 million children and adolescents. Almost 80% of these DALYs avoided concern the reduction of mortality burden of preventable injuries.

## Discussion and Conclusion

The differences in the burden of injury in childhood and adolescence are large, with an enormous gap between the safest countries in Western Europe (the Netherlands and UK) and the relatively unsafe countries (Latvia and Slovenia) in the east. Both differences in premature mortality and disability contribute to the variation in injury burden. In all countries, the highest burden by far is observed among males aged 15-24 years, which is caused by high premature mortality. Skull-brain and spinal cord injury resulted in the highest total YLD due to life-long disability. Superficial injury resulted in the highest short-term disability, due to a high incidence, despite the short course of disability.

The high burden and observed variation of child and adolescence injury in Europe is largely unnecessary, since an abundance of simple and effective intervention strategies are available but underused. As a consequence, large potential health gains can be reached if childhood and adolescence injuries were reduced to the level of current best injury prevention practices in Europe, which we assume is the Netherlands, with the lowest burden of injury. A recent study of WHO supports this assumption by stating that '*the Netherlands and the UK having the lowest child injury mortality rates in the world, Europe has the opportunity to share lessons learnt throughout the region'*[4]. We showed that by reducing mortality and disability of childhood and adolescence injuries to the best current level almost 900 thousand DALYs can be avoided across Europe. This means that each day around 25,000 healthy life years can be gained in the European Union, mainly caused by saving 24 child lives each day. Although the Netherlands represent current best practice, it would be appropriate to note that this should be a floating, improving best practice standard.

This warrants immediate action for injury prevention targeted at high-risk groups and high risk areas. At the European level, males aged 15-24 years are a major high-risk group, since they account for half of the total injury burden in childhood and adolescence (mainly because of traffic accidents and intentional injuries) in all participating countries. In an earlier study in which comparative data on the burden of injury patients in all age categories in Europe was described [9], it was even found that compared to the whole population the highest number of DALYs per 1000 persons is observed in males aged 15-24 years for all age groups. At the country level, specific combinations of external causes and types of injury deserve special attention. The high injury mortality in Latvia, for example, is partly caused by a relatively high mortality rate for suicide and self-inflicted injury for males aged 15-24 years. But most of all, our study draws attention to the enormous gap within Europe between countries in the west and east, as strongly reflected by the fivefold difference in DALYs from child injuries between the Netherlands and Latvia. This inequality already starts among toddlers, extends at school ages and gets further amplified in adolescence. Previous studies have shown that the latter partially could be counteracted by policies aimed at the reduction of excessive drinking in the east[20,21]. But far more action is needed to reduce health inequalities across Europe[22].

The need for immediate action is further stressed, because the reported burden of injury estimates are probably conservative. The burden of injury has been shown to be notably high in comparison to other causes of mortality and loss of health. The GBD estimated that injuries accounted for 16% of DALYs among children and adolescents worldwide[23].

Burden of injury studies are only as good as the weakest link in the chain, which is the epidemiological data[24]. Agenda setting for the collection of good quality epidemiological data is an important issue to emerge from our study. For instance, incidence data for non-admitted ED patients with traffic or intentional injuries was not available in all participating countries. Although this hampered straightforward international comparisons of short-term YLD, its influence is probably modest, since the majority of the injuries of non-admitted ED patients are home and leisure injuries (75%),[9] and their share in the total burden is low (for most countries, less than 2%). More important is that the burden of injury estimates presented in the current study do not picture the impact of injury among children completely. The burden of disabling and life-long sequelae of burn injuries and direct and indirect effects of intoxication were omitted because accurate incidence data were not available. Yet more striking is that psychological consequences of injury were not included, whereas evidence suggests that posttraumatic stress disorder (PTSD) and acute stress disorder are highly prevalent among children hospitalized for injury[25,26].

In addition, underestimations of YLD have probably been reported for specific countries. A good example is Slovenia, where YLD resulting from lifelong disability has been underestimated since information about spinal cord and complex soft tissue injury was largely underestimated in their hospital discharge register.

Furthermore, in the original GBD study that aspired to estimate the total burden of disease worldwide as the sum of the burden of all separate diseases age-weighting was used. With age-weighting the altering levels of dependency with age are taken into account, meaning that years lived at youngest and oldest age is given less weight. Age-weighting has been highly criticized on equity grounds, the absence of empirical foundation and validation, and because the age weights do not convey actual social values[18,19,27,28]. On these grounds, we chose not to apply age-weighting in the current study. However, if age-weighting was applied, it would affect the resulting burden of injury estimates, since mortality among adolescents, which causes the lion's share of DALYs lost, would get even more emphasis compared to mortality among young children.

As shown in this article, unintentional and intentional injuries in childhood and adolescence cause a high disability and mortality burden in Europe. In all developmental stages large inequalities between west and east are observed. This study has shown the huge potential benefits that can be realized by implementing interventions that are proven ways to reduce both the likelihood and severity of injury. However, according to the WHO injuries are remarkably neglected, compared with the attention devoted to research and policy for other leading causes of DALYs worldwide[4]. Our children deserve better and need improved protection now.

## Competing interests

The authors declare that they have no competing interests.

## Authors' contributions

SP had full access to all of the data in the study and takes responsibility for the integrity of the data and the accuracy of the data analysis. Furthermore, she is partly responsible for the study concept and design and drafted the manuscript. EB was responsible for the study supervision, the study concept and design of the study and drafted the manuscript. JH and HT were involved in the analysis and interpretation of the data. JH, HT and MB gave a critical revision for important intellectual content of the manuscript. All authors read and approved the final manuscript.

## Pre-publication history

The pre-publication history for this paper can be accessed here:

http://www.biomedcentral.com/1471-2458/10/45/prepub

## Supplementary Material

Additional file 1 **Appendix A**. Overview of disability weights and duration of health state for injuries in the GBD^a^ Click here for file
