# Supplementary material for: Burden of injury in childhood and adolescence in 8 European countries
Source: BMC Public Health. 2010 Jan 29;10:45. doi: 10.1186/1471-2458-10-45 (PMC2824737; doi:10.1186/1471-2458-10-45)
Supplement: Additional file 1 — Appendix A. Overview of disability weights and duration of health state for injuries in the GBDa [file 1471-2458-10-45-S1.DOC]

Appendix A. Overview of disability weights and duration of health state for injuries in the GBD^a^

| **Injury groups** | **GBD disability weight** | **Duration of disability (years)** |
| --- | --- | --- |
|
| 1. Concussion | 0.020^b^ | — |
| 2. Other skull-brain injury | ST^c^ 0.359 — 0.431 LL^d^ 0.350^e^ | 0.107 — LL15% ^e^ |
| 3. Open wound head | 0.108 | 0.024 |
| 4. Eye injury | 0.004^b^ | - |
| 5. Fracture facial bones | 0.233 | 0.118 |
| 6. Open wound face | 0.108 | 0.024 |
| 7. Vertebral column fractures / dislocations / sprain / strain | 0.266 | 0.140 |
| 8. Whiplash, neck sprain, distortion of cervical spine | 0.094^b^ | — |
| 9. Spinal cord injury | 0.725 | 100%LL |
| 10. Internal organ injury | 0.208 | 0.042 |
| 11. Fracture rib / sternum | 0.199 | 0.115 |
| 12. Fracture of clavicle / scapula | 0.153–0.137 | 0.112 |
| 13. Fracture of upper arm | 0.153–0.137 | 0.112 |
| 14. Fracture of elbow / forearm | 0.153–0.137 | 0.112 |
| 15. Fracture of wrist (including carpal bones) | 0.100 | 0.112 |
| 16. Fracture of hand / fingers | 0.100 | 0.070 |
| 17. Dislocation / sprain / strain shoulder / elbow | 0.074 | 0.035 |
| 18. Dislocation / sprain / strain wrist / hand / fingers | 0.064 | 0.035 |
| 19. Injury of nerves arm/hand | 0.064 | 100%LL |
| 20. Amputation upper extremity | 0.102 — 0.165 | 100%LL |
| 21. Fracture of pelvis | 0.247 | 0.126 |
| 22. Fracture of hip | ST 0.372 — LL 0.272 | 0.139 — LL5% ^e^ |
| 23. Fracture of femur shaft | ST 0.372 — LL 0.272 | 0.139 — LL5% ^e^ |
| 24. Fracture of knee / lower leg | 0.196 | 0.090 |
| 25. Fracture of ankle | 0.196 | 0.096 |
| 26. Fracture of foot | 0.077 | 0.073 |
| 27. Dislocation / sprain / strain of knee | 0.064 | 0.035 |
| 28. Dislocation / sprain / strain of ankle / foot | 0.064 | 0.035 |
| 29. Dislocation / sprain / strain of hip | 0.074 | 0.035 |
| 30. Injury of nerves leg/foot | 0.064 | 100%LL |
| 31. Amputation lower extremity | 0.300 | 100%LL |
| 32. Superficial injury (including contusions) | 0.005^b^ | — |
| 33. Open wounds | 0.108 | 0.024 |

^a^ GBD = Global Burden of Disease[5]

^b^ Dutch disability weights[16]

^c^ ST = Short-term.

^d^ LL = Lifelong.

^e^ A proportion of patients has lifelong sequelae. The other patients have short-term disability.
